# The 2018 periodontitis case definition improves accuracy performance of full-mouth partial diagnostic protocols

**DOI:** 10.1038/s41598-020-63700-6

**Published:** 2020-04-27

**Authors:** João Botelho, Vanessa Machado, Luís Proença, José João Mendes

**Affiliations:** 1 Periodontology Department, Clinical Research Unit (CRU), Centro de Investigação Interdisciplinar Egas Moniz (CiiEM), Instituto Universitário Egas Moniz (IUEM), Almada, Portugal; 2 Clinical Research Unit (CRU), CiiEM, IUEM, Almada, Portugal; 3 Quantitative Methods for Health Research (MQIS), CiiEM, IUEM, Almada, Portugal

**Keywords:** Dental diseases, Epidemiology, Translational research, Outcomes research

## Abstract

We aimed to compare the accuracy performance of the new 2018 periodontitis case definition by the European Federation of Periodontology (EFP)/ American Association of Periodontology (AAP) with Centers for Disease Control (CDC)/AAP 2012 in full-mouth partial recording protocols (PRP). Retrospective data from NHANES 2011-2012 and 2013-2014 were analyzed. For each case definition, full-mouth diagnostic was defined as the reference standard. Patients were diagnosed for the presence of periodontitis and staging for each PRP. Sensitivity, specificity, accuracy and precision, through several indicators, were determined. Performance measurement was assessed through binary and multiclass ROC/AUC analyses. Our performance analysis shows that the new 2018 classification outperforms the 2012 classification regarding the diagnosis and staging of periodontitis on full-mouth PRPs. This recent case definition has strengthened the utility of PRPs and its improvements certainly explain the observed findings. Also, our findings contribute to the reliability of PRPs and its use in future worldwide epidemiological surveys.

## Introduction

The constant pursuit of improved protocols on how periodontal diseases are diagnosed represents the disease complexity and a scientific community aiming for valid and accurate systems. In 2018, a world consensus from the European Federation of Periodontology (EFP) and the American Academy of Periodontology (AAP) provided a new classification of periodontal diseases^1^. This new classification revised unsettled points from previous classification^2^, showing a reliable capacity to reflect patients’ characteristics, disease evolution and tooth loss^3^.

In 2012, the Centers for Disease Control (CDC) with the AAP proposed a standard case definition for surveillance periodontitis based on measurements of periodontal probing depth (PPD) and clinical attachment loss (CAL) at interproximal sites^4^. Ever since this case definition has been widely accepted and applied both in epidemiological and clinical research. In the new 2018 classification, in addition to the interproximal sites, mid-buccal and mid-lingual sites were also considered^5^. Comprehensively, this addition reinforces this new classification with a potential improved ability to transmit the entire periodontal condition.

Furthermore, the 2012 and 2018 case definitions demand circumferential full-mouth inspection, which in large surveys and epidemiological studies it is often difficult to conduct, and time and labour intensive^6–8^. In this sense, several partial recording protocols (PRP) have been proposed for a full and partial mouth, though full-mouth PRPs present much less biasing potential^6,9–11^. Therefore, considering the improvements in the new 2018 case definition, it is reasonable to consider that it could contribute to more reliable and accurate PRPs using this diagnostic framework.

The present study aimed to compare how the new 2018 EFP/AAP classification performs in full-mouth PRPs for presence and staging of periodontitis, in comparison with the 2012 CDC/AAP.

## Methods

### Source of data and study population

NHANES 2011-2012 and 2013–2014 data were obtained through the CDC and Prevention National Center for Health Statistics (NCHS) website at https://www.cdc.gov/nchs/nhanes/index.htm. The present study was deemed exempt from review by the Egas Moniz Ethics Committee.

It focuses on participants who underwent periodontal health status evaluation. Oral health data collection protocols were approved by the CDC, NCHS Research Ethics Review Board, Atlanta (USA), and all participants gave written informed consent. All the examinations were conducted in a mobile examination centre. In these datasets, participants younger than 30 years of age were excluded due to reason explained elsewhere (see in detail in^12^). This study follows the Standards for Reporting of Diagnostic Accuracy Studies (STARD) statement^13^.

### Eligibility criteria and periodontal examination

Exclusion criteria accounted for participants with medical exclusion from periodontal exam, non-complete periodontal status and edentulous. Periodontal examination consisted of a circumferential assessment of PPD and CAL around each tooth for all teeth. Third molars were excluded from the analysis.

### Periodontitis case definition

For this study, we used the 2018 World Workshop EFP/AAP consensus^5^ and the 2012 CDC/AAP case definition^4^.

In the 2018 EFP/AAP case definition, a participant was a periodontitis case if: interdental CAL ≥ 2 non‐adjacent teeth, or Buccal or Oral CAL ≥ 3 mm with PPD > 3 mm is detectable at ≥2 teeth. Then, periodontitis staging was defined according to presence and stage^5^. For the staging, interdental CAL at the site of greatest loss of 1–2, 3–4 and ≥5 mm were considered as mild (stage 1), moderate (stage 2), and severe (stage 3 and stage 4), respectively^5^.

In the 2012 CDC/AAP case definition, a participant was a case of: *Mild periodontitis* − 2 or more interproximal sites with CAL ≥ 3 mm, and 2 or more interproximal sites with PPD ≥ 4 mm (not on the same tooth) or one site with PPD ≥ 5 mm; *Moderate periodontitis* − 2 or more interproximal sites with CAL ≥ 4 mm (not on the same tooth) or 2 or more interproximal sites with PPD ≥ 5 mm, also not on the same tooth); *Severe periodontitis* - the presence of 2 or more interproximal sites with CAL ≥ 6 mm (not on the same tooth) and 1 or more interproximal site(s) with PPD ≥ 5 mm; *No periodontitis* - no evidence of mild, moderate, or severe periodontitis^4^.

### Full-mouth partial recording protocols

For this study, 6-sites PRPs selected were: 1) “Ramfjord teeth”^14^ on 6-site circumferential inspection (mesio-buccal [MB], mid-buccal [B], distal-buccal [DB], mesio-lingual [ML], mid-lingual [L], distal-lingual [DL]) – right maxillary first molar (16), left maxillary central incisor (21), left maxillary first premolar (24), left mandibular first molar (36), right mandibular central incisor (41) and right mandibular first premolar (44); 2) Community Periodontal Index of Treatment Needs (CPITN) teeth^15^ on 6-site circumferential inspection – right maxillary first and second molar (16,17), right maxillary central incisor (11), left maxillary first and second molar (26,27), left mandibular first and second molar (36,37), left mandibular central incisor (31) and right mandibular first and second molar (46,47). 3) MB–B measurements in all teeth; 4) MB–B–DB measurements in all teeth; 5) MB–B–DL measurements in all teeth. We decided to use these indexes based on the high bias (Ramfjord and CPITN) and low bias (MB-B, MB-B-DB, MB-B-DL) previously reported^9–11^.

### Data management, test methods and analysis

Data were uploaded through SAS Universal Viewer for Windows and handled with Microsoft Office (MO) Excel. For each periodontal case definition, specific MO Excel datasets were derived in order to formulate appropriate algorithms.

In the performance analysis, full-mouth diagnosis was used as the standard reference for each case definition because it represents entirely the periodontal status. To test the index performance, we started by computing the final diagnosis into two variables according to the presence of disease (coded: 0-no, 1-yes) and the staging (coded: 0- non periodontitis, 1-mild, 2-moderate, 3-severe). Then, contingency tables were used to calculate true positive (TP), true negative (TN), false positive (FP) and false negative (FN) values. From this, sensitivity, specificity, accuracy and precision, through several indicators, were determined (Table 1)^16^. Also, Diagnostic Odds Ratio (DOR) and the respective standard error (SE) and 95% confidence interval (95% CI) were estimated. Performance measurement was assessed through binary and multiclass Area Under the Curve (AUC), through Receiver Operating Characteristics (ROC) analysis. For AUC/ROC analysis, we used the R package “plotROC”^17^ (by means of “roc” and “multiclass.roc” functions). The evolution of the periodontal status, from the 2012 CDC/AAP to the 2018 EFP/AAP case definition, was assessed through an alluvial diagram using https://app.rawgraphs.io/. Data were analysed as originally recorded, without missing data handling.

**Table 1:** Diagnostic performance indicators used in the comparative analysis.

Sensitivity (True positive rate)	TP/(TP + FN)	Proportion positive test results among diseased
Specificity (True negative rate)	TN/(TN + FP)	Proportion negative test results among the “healthy”
Accuracy	(TP + TN)/(TP + TN + FP + FN)	Proportion of correctly identified subjects
Precision - Positive Predictive Values (PPV)	TP/(TP + FP)	—
Youden’s index	Sensitivity + Specificity − 1	Measures the performance of a dichotomous diagnostic test
DOR	(TP/FN)/(FP/TN)	Ratio of the odds of positivity in disease relative to the odds of positivity in the non-diseased
DOR (95% CI)	95% CI = log DOR ± 1.96SE(log DOR), where $SE(logDOR)=\sqrt{\frac{1}{TP}+\frac{1}{TN}+\frac{1}{FP}+\frac{1}{FN}}$	—
F1 Score	2TP/(2TP+FP+FN)	Harmonic mean of precision and sensitivity
Matthews Correlation Coefficient (MCC)	(TP x TN - FP x FN)/$\sqrt{(({\rm{TP}}+{\rm{FP}}){\rm{x}}({\rm{TP}}+{\rm{FN}}){\rm{x}}({\rm{TN}}+{\rm{FP}}){\rm{x}}({\rm{TN}}+{\rm{FN}})}$	Measure of quality of binary classifications

95% CI – 95% Confidence Interval; DOR – Diagnostic Odds Ratio; FN – False Negative, FP – False Positive; SE – Standard Error; TN – True Negative; TP –True Positive. Adapted from 16.

## Results

### Population

**Figure 1:**
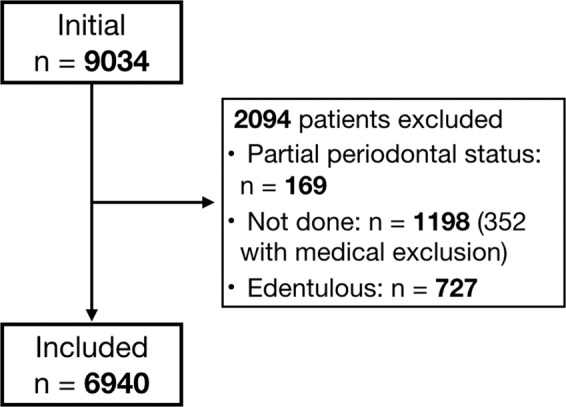
Flow of participants.

From an initial sample of 9,034 individuals, eligibility criteria were applied resulting in a final sample of 6,940 participants (Fig. 1). The baseline demographic, clinical characteristics of participants and distribution of severity of disease in the target condition were fully described elsewhere (for more details see^12,18^).

### Accuracy performance for the presence of periodontitis

Regarding the presence of periodontitis, the 2018 case definition outperforms the sensitivity analyses of all PRPs, though the 2012 case definition presents excellent specificity results (Table 2). Because of the high specificity rates, the 2012 classification shows higher precision and DOR values. However, when looking for the remaining performance indexes, the 2018 classification clearly outperforms the 2012 one, in particular, the AUC values.

**Table 2:** Performance assessment of 2018 EFP/AAP and 2012 CDC/AAP case definitions on full-mouth PRPs. Measures presented as percentage with the correspondent 95% CI.

	Ramfjord Teeth	CPITN Teeth	MB-B	MB-B-DB	MB-B-DL
**Sensitivity**					
2018 EFP/AAP	63.2 (62.0–64.3)	87.1 (86.3–87.9)	69.3 (68.2–70.4)	79.1 (78.1–80.0)	84.9 (84.0–85.7)
2012 CDC/AAP	40.5 (39.4–41.7)	41.5 (40.4–42.7)	49.6 (48.4–50.7)	64.0 (62.9–65.1)	72.9 (71.9–74.0)
**Specificity**					
2018 EFP/AAP	95.2 (94.7–95.7)	78.5 (77.5–79.5)	93.0 (92.4–93.6)	91.0 (90.4–91.7)	90.8 (90.2–91.5)
2012 CDC/AAP	99.9 (99.8–100.0)	99.8 (99.7–99.9)	99.9 (99.8–100.0)	99.4 (99.2–99.6)	99.1 (98.8–99.3)
**Accuracy**					
2018 EFP/AAP	85.8 (85.0–86.6)	81.0 (80.1–82.0)	86.1 (85.3–86.9)	87.5 (86.7–88.3)	89.1 (88.4–89.8)
2012 CDC/AAP	65.9 (64.7–67.0)	66.4 (65.3–67.5)	71.1 (70.0–72.1)	79.1 (78.2–80.1)	84.1 (83.2–85.0)
**Precision**					
2018 EFP/AAP	84.5 (83.7–85.4)	62.7 (61.6–63.9)	80.5 (79.6–81.5)	78.5 (77.6–79.5)	79.4 (78.4–80.3)
2012 CDC/AAP	99.8 (99.6–99.9)	99.6 (99.5–99.8)	99.8 (99.7–99.9)	99.3 (99.1–99.5)	99 (98.8–99.3)
**Youden’s index**					
2018 EFP/AAP	58.4 (57.2–59.5)	65.6 (64.5–66.8)	62.3 (61.2–63.5)	70.1 (69.0–71.2)	75.7 (74.7–76.7)
2012 CDC/AAP	40.4 (39.2–41.5)	41.3 (40.2–42.5)	49.4 (48.3–50.6)	63.4 (62.3–64.6)	72.0 (70.9–73.0)
**Diagnostic Odds Ratio (DOR)**					
2018 EFP/AAP	1.53 (1.37–1.69)	1.39 (1.25–1.54)	1.48 (1.34–1.62)	1.58 (1.44–1.73)	1.75 (1.59–1.9)
2012 CDC/AAP	2.7 (1.72–3.69)	2.54 (1.74–3.35)	2.86 (1.88–3.84)	2.49 (2.01–2.97)	2.45 (2.07–2.83)
**F1 Score**					
2018 EFP/AAP	72.3 (71.3–73.4)	72.9 (71.9–74.0)	74.5 (73.5–75.5)	78.8 (77.8–79.8)	82.0 (81.1–82.9)
2012 CDC/AAP	57.6 (56.5–58.8)	58.6 (57.5–59.8)	66.2 (65.1–67.4)	77.9 (76.9–78.8)	84.0 (83.1–84.9)
**Matthews Correlation Coefficient (MCC)**					
2018 EFP/AAP	64.2 (63.1–65.4)	60.8 (59.7–62.0)	65.3 (64.2–66.5)	70.0 (68.9–71.0)	74.3 (73.3–75.3)
2012 CDC/AAP	47.3 (46.1–48.4)	47.9 (46.8–49.1)	54.2 (53.0–55.4)	65.0 (63.9–66.1)	72.1 (71.1–73.2)
**AUC (within ROC analysis)**					
2018 EFP/AAP	71.2 (70.1–72.3)	82.8 (81.9–83.7)	81.2 (80.3–82.1)	85.1 (84.2–85.9)	87.9 (87.1–88.6)
2012 CDC/AAP	70.2 (69.1–71.3)	70.7 (69.6–71.7)	74.7 (73.7–75.7)	81.7 (80.8–82.6)	86.0 (85.2–86.8)

95% CI – 95% Confidence Interval; AUC – Area Under the Curve; AAP – American Academy of Periodontology; B – Mid-buccal; CDC – Centers for Diseases Control; CPITN – Community Periodontal Index of Treatment Needs; DB – distal-buccal; DL – distal-lingual; EFP – European Federation of Periodontology; MB – Mesial-buccal; ROC – Receiver Operating Characteristic.

### Accuracy performance for staging

Multiclass ROC analyses, through AUC values, show that 2018 EFP/AAP outperforms 2012 CDC/AAP in all PRPs (Table 3). In terms of precision, 2012 CDC/AAP has excellent predictive values for non periodontitis patients and very low values for mild periodontitis patients. The 2018 EFP/AAP improved the precision for mild periodontitis patients in all PRPs, though for moderate and severe cases the results are similar to the 2012 CDC/AAP. Interestingly, the 2018 case definition has improved remarkably the precision performance of Ramfjord and CPITN teeth.

**Table 3:** Staging ROC/AUC values and precision of 2018 EFP/AAP and 2012 CDC/AAP case definitions on full-mouth PRPs.

AUC (95% CI)	Ramfjord Teeth	CPITN Teeth	MB-B	MB-B-DB	MB-B-DL
2018 EFP/AAP	79.1 (78.1–80.0)	86.6 (85.8–87.4)	80.9 (79.9–81.8)	86.4 (85.6–87.2)	89.5 (88.8–90.3)
2012 CDC/AAP	75.7 (74.7–76.7)	74.5 (73.5–75.5)	79.8 (78.8–80.7)	84.6 (83.8–85.5)	87.6 (86.8–88.3)
**2018 EFP/AAP Staging Precision (95% CI)**					
Non periodontitis	95.2 (94.7–95.7)	78.5 (77.5–79.5)	93.0 (92.4–93.6)	91.0 (90.4–91.7)	90.8 (90.2–91.5)
Mild	37.2 (36.1–38.4)	78.0 (77.0–78.9)	47.6 (46.4–48.8)	57.8 (56.7–59.0)	64.7 (63.6–65.9)
Moderate	37.8 (36.6–38.9)	73.5 (72.4–74.5)	36.0 (34.8–37.1)	60.8 (59.6–61.9)	72.9 (71.9–74.0)
Severe	43.0 (41.8–44.2)	66.0 (64.9–67.1)	41.5 (40.3–42.6)	63.6 (62.4–64.7)	72.5 (71.5–73.6)
**2012 CDC/AAP Staging Precision (95% CI)**					
Non periodontitis	99.9 (99.8–100)	99.8 (99.7–99.9)	99.9 (99.8–100)	99.4 (99.2–99.6)	99.1 (98.8–99.3)
Mild	0.8 (0.6–1.0)	2.5 (2.2–2.9)	1.1 (0.8–1.3)	4.4 (3.9–4.9)	6.7 (6.1–7.3)
Moderate	34.9 (33.8–36.1)	35.4 (34.3–36.5)	48.1 (46.9–49.3)	68.7 (67.6–69.8)	83.3 (82.4–84.1)
Severe	26.6 (25.5–27.6)	10.3 (9.6–11.0)	37.7 (36.5–38.8)	61.2 (60.1–62.4)	73.0 (72.0–74.1)

95% CI – 95% Confidence Interval; AUC – Area Under the Curve; AAP – American Academy of Periodontology; B – Mid-buccal; CDC – Centers for Diseases Control; CPITN – Community Periodontal Index of Treatment Needs; DB – distal-buccal; DL – distal-lingual; EFP – European Federation of Periodontology; MB – Mesial-buccal.

### Comparison of classifications

**Figure 2:**
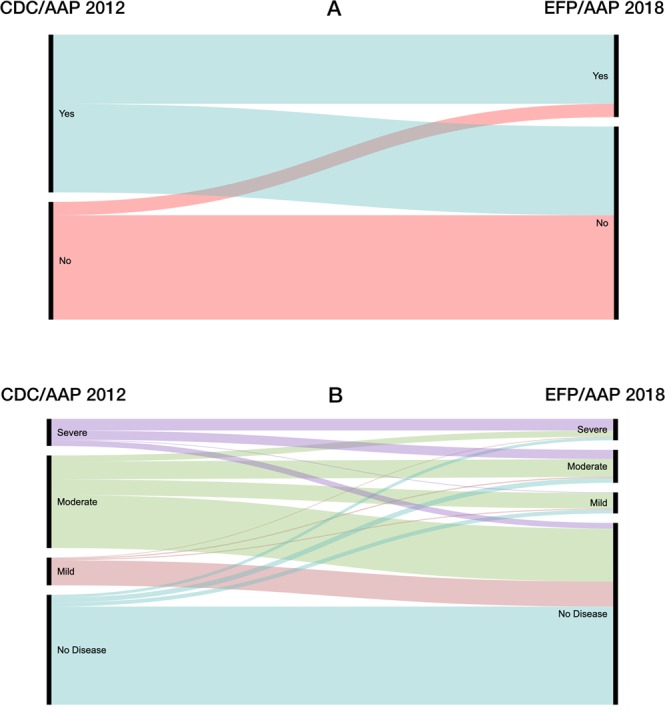
Periodontal diagnosis evolution from the 2012 CDC/AAP (left block) to the 2018 EFP/AAP case definition (right block) according to the presence of periodontitis (**A**) and the staging (**B**). Colored stream fields between the blocks depict variations over time.

We further assessed how patients classified according to the 2012 classification were re‐classified in the 2018 classification (Fig. 2). Most patients with periodontitis were re‐classified in non periodontitis cases. Mostly, moderate and mild periodontitis patients were re-classified as non periodontitis. Also, a set of non periodontitis patients had their status updated to periodontitis, and several patients had their severity downgraded or even diagnosed as non periodontitis.

## Discussion

In our investigation, we hypothesized that the 2018 EFP/AAP periodontal classification could improve the performance on PRPs comparing to the 2012 CDC/AAP classification. To examine this hypothesis, we compiled a significant dataset from the NHANES between 2011 and 2014 and we performed several diagnostic performance indicators in a comparative analysis. Our results confirmed that the new 2018 classification outperforms the 2012 classification regarding the diagnosis of periodontitis and its staging on full-mouth PRPs.

Our findings have significant wide implications. (1) From a perspective of population-based surveillance of periodontitis, the 2018 case definition has strengthened the utility of PRPs comparing to the 2012 classification. (2) The 2012 case definition has excellent specificity properties. (3) The comparison of classifications provided interesting re-classification findings. (4) Considering the challenges of performing full-mouth protocols in large studies, the 2018 EFP/AAP classification on full-mouth PRPs might contribute to enhance the number of population-based surveillance studies on periodontal diseases.

The inclusion of central surfaces (mid-buccal and mid-lingual sites) in the 2018 case definition^1,5^ has endowed it with a holistic view of the periodontal situation. In other words, by considering all circumferential sites we increase the likelihood of correctly diagnosing periodontitis, rather than the 2012 classification that only uses interproximal locations (maximum of four sites). Moreover, both case definitions evidenced cases whose final diagnosis did not coincide, with several periodontitis patients in the 2012 classification being re-classified as non periodontitis patients in the 2018 case definition.

On the one hand, the new 2018 EFP/AAP case definition is a reliable tool in depicting patients’ characteristics, disease progression and tooth loss^3^. On the other hand, our findings emphasize its reliability on future epidemiological studies using PRPs, considering that more surveys are warranted to improve surveillance of periodontitis, a pandemic disease with worldwide prevalence and worrisome socio-economic impact^19–24^.

A periodontal diseases surveillance system has intrinsic limitations, in particular, time, number of examiners and complexity of the measurement tool^25^. Therefore, we consider relevant to seek reliable alternatives with current case definitions for the purpose of minimizing these limitations. Regarding the complexity of the measurement strategy, the challenge of the number of teeth and sites to be examined were addressed through the tested indexes. In indexes with a lower number of teeth, the current 2018 case definition endowed CPITN has a more reliable tool in both detecting and staging periodontitis, comparing to the 2012 scenario. For the “Ramfjord teeth”, the 2018 classification provided slight surveillance improvements, though it was the index with less favorable performance. While the indexes with all teeth but with a lower number of sites, the MB-B-DL and MB-B-DB sites had very pleasant results for both diagnosis and staging of periodontitis, unlike the MB-B sites. Interestingly, these results are in agreement with past studies on previous case definitions showing the bias potential of “Ramfjord teeth” and MB-B approaches to estimate the prevalence of periodontitis^9–11^. Besides, several studies have shown excellent predictive results of MB-B-DL and MB-B-DB for PD and CAL periodontal measures^6,9,11^. A possible explanation for the better performance of these three-sites approaches relies on the fact that they encompass the interproximal sites and one central face of all teeth, which provides them with a more comprehensive surveillance ability. However, a possible shortcoming of these three-sites indexes is that we only reduce the periodontal inspection by the halved, though from the epidemiological perspective can be very significative. Henceforth, these full-mouth three-sites PRPs might be of high epidemiological relevance, considering the requirements of the surveillance surveys in periodontal diseases^26^.

To the best of our knowledge, this is the first study examining the performance of the new 2018 classification on PRPs and its epidemiological potential. Previously, data from the NHANES 2009–2010 was used to test several full-mouth and half-mouth PRPs performance on 2012 CDC/AAP case definition^6,10^. Due to the fact that the 2012 classification only accounts for interproximal sites,the authors did not include central surfaces, hence, these results are not comparable to our findings. Similarly to our results, MB-DB and MB-DL presented the most promising results^10^.

This study has strengths and limitations worth mentioning. In contrast to NHANES III that used half-mouth data^6^, full-mouth values were provided minimizing the underestimation of periodontitis in these patients. Also, the dataset is originated from a very significant national health survey, with substantial representativeness and generalizability. Furthermore, measures of interest were assessed by trained and calibrated examiners and the most up-to-date definitions of periodontitis were used making these results current and of high scientific interest, though having multiple examiners may result in variability in the analysis and determination of the stage. Nevertheless, despite AUC, there is high variability of performance indicators that may contribute to less certainty in the interpretation of the results. Additionally, these indicators were developed for less complex clinical diagnostic tests and multiclass assessment on staging accuracy has limited analyses available. Also, we were unable to assess how the disease level influences PRP performance, though this was already reported^27^. From these results, the advantages of applying this case definition on full-mouth PRPs might be the decrease of time and effort invested in diagnosing large representative samples. In particular, the MB-B-DL approach seems to be the PRP with the most potential for prevalence and staging purposes. Interestingly, there apparent a low difference in the therapeutic attitude according to the staging with the new classification compared to the old one, though this was recently addressed ().Future studies should investigate the impact of employing this type of PRPs on epidemiologic settings and how the variation of disease prevalence could affect the performance of such indexes.

## Conclusion

The new 2018 classification outperforms the 2012 classification regarding the diagnosis and staging of periodontitis on full-mouth PRPs. This recent case definition has strengthened the utility of PRPs and its improvements certainly explain the observed findings. Also, our findings contribute to the reliability of PRPs and its use in future epidemiological surveys.

## Supplementary information

Supplementary Information.

## Data Availability

10.5281/zenodo.3581071
